# Smoking and Adverse Outcomes in Patients With CKD: The Study of Heart and Renal Protection (SHARP)

**DOI:** 10.1053/j.ajkd.2016.02.052

**Published:** 2016-09

**Authors:** Natalie Staplin, Richard Haynes, William G. Herrington, Christina Reith, Alan Cass, Bengt Fellström, Lixin Jiang, Bertram L. Kasiske, Vera Krane, Adeera Levin, Robert Walker, Christoph Wanner, David C. Wheeler, Martin J. Landray, Colin Baigent, Jonathan Emberson, SHARP Collaborative Group, Colin Baigent, Martin J. Landray, Christina Reith, Jonathan Emberson, David C. Wheeler, Charles Tomson, Christoph Wanner, Vera Krane, Alan Cass, Jonathan Craig, Bruce Neal, Lixin Jiang, Lai Seong Hooi, Adeera Levin, Lawrence Agodoa, Mike Gaziano, Bertram L. Kasiske, Robert Walker, Ziad A. Massy, Bo Feldt-Rasmussen, Udom Krairittichai, Vuddidhej Ophascharoensuk, Bengt Fellström, Hallvard Holdaas, Vladimir Tesar, Andrzej Wiecek, Diederick Grobbee, Dick de Zeeuw, Carola Grönhagen-Riska, Tanaji Dasgupta, David Lewis, William G. Herrington, Marion Mafham, William Majoni, Karl Wallendszus, Richard Grimm, Terje Pedersen, Jonathan Tobert, Jane Armitage, Alex Baxter, Christopher Bray, Yiping Chen, Zhengming Chen, Michael Hill, Carol Knott, Sarah Parish, David Simpson, Peter Sleight, Alan Young, Rory Collins

**Affiliations:** 1 Clinical Trial Service Unit and Epidemiological Studies Unit, Nuffield Department of Population Health, University of Oxford, Oxford, United Kingdom; 2 Menzies Institute, Darwin, Australia; 3 University Hospital, Uppsala, Sweden; 4 China Oxford Centre for International Health Research, Fuwai Hospital, Beijing, China; 5 University of Minnesota, Minneapolis, MN; 6 Division of Nephrology, University of Wuerzberg, Wuerzberg, Germany; 7 University of British Columbia, Vancouver, BC, Canada; 8 University of Otago, Dunedin, New Zealand; 9 University College London, London, United Kingdom

**Keywords:** Cigarette smoking, tobacco, chronic kidney disease (CKD), vascular morbidity, end-stage renal disease (ESRD), risk factor, cause-specific mortality, vascular events, cancer, estimated glomerular filtration rate (eGFR), disease progression, Study of Heart and Renal Protection (SHARP)

## Abstract

**Background:**

The absolute and relative importance of smoking to vascular and nonvascular outcomes in people with chronic kidney disease (CKD), as well its relevance to kidney disease progression, is uncertain.

**Study Design:**

Observational study.

**Setting & Participants:**

9,270 participants with CKD enrolled in SHARP.

**Predictor:**

Baseline smoking status (current, former, and never).

**Outcomes:**

Vascular events, site-specific cancer, ESRD, rate of change in estimated glomerular filtration rate (eGFR), and cause-specific mortality.

**Results:**

At baseline, 1,243 (13%) participants were current smokers (median consumption, 10 cigarettes/day); 3,272 (35%), former smokers; and 4,755 (51%), never smokers. Median follow-up was 4.9 years. Vascular event rates were 36% higher for current than never smokers (2,317 events; relative risk [RR], 1.36; 95% CI, 1.19-1.55), reflecting increases in both atherosclerotic (RR, 1.49; 95% CI, 1.26-1.76) and nonatherosclerotic (RR, 1.25; 95% CI, 1.05-1.50) events. Cancer was 37% higher among current smokers (632 events; RR, 1.37; 95% CI, 1.07-1.76), with the biggest RRs for lung (RR, 9.31; 95% CI, 4.37-19.83) and upper aerodigestive tract (RR, 4.87; 95% CI, 2.10-11.32) cancers. For 6,245 patients not receiving dialysis at baseline, ESRD incidence did not differ significantly between current and never smokers (2,141 events; RR, 1.02; 95% CI, 0.89-1.17), nor did estimated rate of change in eGFR (current smokers, −1.77 ± 0.14 [SE]; never smokers, −1.70 ± 0.07 mL/min/1.73 m^2^ per year). All-cause mortality was 48% higher among current smokers (2,257 events; RR, 1.48; 95% CI, 1.30-1.70), with significant increases in vascular (RR, 1.35; 95% CI, 1.07-1.69) and nonvascular (RR, 1.60; 95% CI, 1.34-1.91) causes of death, especially cancer (RR, 2.32; 95% CI, 1.58-3.40) and respiratory (RR, 2.25; 95% CI, 1.51-3.35) mortality.

**Limitations:**

Smoking status not assessed during follow-up.

**Conclusions:**

In this study of patients with CKD, smoking significantly increased the risks for vascular and nonvascular morbidity and mortality, but was not associated with kidney disease progression. The associations with vascular and neoplastic disease are in keeping with those observed in the general population and are likely modifiable by cessation.

Editorial, p. 338

In 2010, it was estimated that smoking tobacco resulted in about 4.5 million male and 2 million female deaths globally.^1^ Smoking was the leading global cause of disability in men and fourth most important cause in women and was ranked in the top 5 causes of disease and death in every region of the world except for parts of sub-Saharan Africa.^1^ Smoking tobacco increases the risks for a wide range of chronic conditions, including cancer,^2^, ^3^, ^4^, ^5^ cardiovascular diseases^2^, ^3^, ^4^, ^5^ (coronary heart disease, stroke, and heart failure), respiratory diseases,^2^, ^3^, ^4^ and chronic kidney disease (CKD).^5^, ^6^

Patients with CKD have substantially elevated risks for cancer,^7^ cardiovascular disease,^8^ and progressive loss of kidney function^9^; therefore, smoking might be expected to be particularly hazardous for such patients. However, few epidemiologic studies have examined the effects of smoking directly in populations of patients with CKD. In the largest previous study (in which 3,941 dialysis patients were followed up for a mean of 2.2 years), current smokers had a 59% increased risk for new heart failure and 37% increased risk for death when compared with never smokers,^10^ but there was no significant excess risk for ischemic heart disease or cerebrovascular disease (albeit based on only 232 and 64 events, respectively).^10^

The Study of Heart and Renal Protection (SHARP) was a randomized trial of simvastatin plus ezetimibe versus placebo among 9,270 patients with CKD.^11^ It is well placed to address the existing uncertainties about the effects of smoking in patients with CKD. More than twice as large as previous studies,^12^, ^13^ SHARP has carefully phenotyped participants at baseline, with systematic recording of smoking quantity, prior diseases, and laboratory measurements. In addition, all relevant outcomes were systematically adjudicated so that reliable information is available for cause-specific mortality (>2,200 cases), atherosclerotic and nonatherosclerotic vascular events (>2,300 cases), site-specific cancer (∼900 cases), and progression of CKD (including serial creatinine measurements and >2,100 patients reaching end-stage renal disease [ESRD]). We therefore undertook these analyses to investigate the relevance of smoking to major morbidity and mortality in a large cohort of patients with moderate to advanced CKD.

## Methods

### Study Overview

From June 2003 through June 2006, a total of 9,270 participants with CKD were enrolled into SHARP and randomly assigned to ezetimibe/simvastatin combination therapy versus placebo.^11^, ^14^ The main trial methods and results from the randomized comparisons have been published previously.^11^, ^14^ Procedures relevant to the current analyses are summarized next. Ethics approval was obtained from all study sites prior to enrollment.

### Study Participants and Baseline Assessment

For the purpose of the current observational analyses, baseline information refers to information that was recorded at (or in some cases shortly before) the point at which participants were randomly assigned to treatment with simvastatin plus ezetimibe versus placebo. Individuals 40 years or older were eligible to participate if they were receiving maintenance dialysis or had CKD with more than 1 previous measurement of serum or plasma creatinine of at least 1.7 mg/dL (150 μmol/L) in men or 1.5 mg/dL (130 μmol/L) in women. Individuals with known prior myocardial infarction, coronary revascularization, or cancer (except for nonmelanoma skin cancer) were excluded. All participants provided written informed consent. Recorded baseline information included sociodemographic characteristics (including ethnicity and highest educational achievement), anthropometric measurements, self-reported diagnoses of previous diseases, current medication, and lifestyle characteristics (including alcohol consumption and smoking status). Information for smoking status included whether the participant was currently or had previously ever been a regular smoker of cigarettes and, for current smokers, the number of cigarettes smoked on a usual day. Baseline nonfasting blood and urine samples for central laboratory measurement were also collected. Creatinine was measured using a kinetic alkaline picrate method, and glomerular filtration rate (GFR) was estimated using the 4-variable MDRD (Modification of Diet in Renal Disease) Study equation.^15^

### Follow-up Procedures and Study Outcomes

Participants were to be seen in person at 2 months and then every 6 months throughout the study, with additional follow-up procedures (eg, by telephone) arranged when necessary. All participants were to be followed up for at least 4 years. At each visit, information for all serious adverse events (including all hospitalizations, new diagnoses of cancer, and initiation of renal replacement therapy) was sought and further documentation was collected on outcomes of interest (including all vascular events, cancer, initiation of renal replacement therapy, and all deaths). This information was sent to the international coordinating center for central adjudication by trained clinicians, in accordance with prespecified definitions. For the purpose of the present analyses, we defined the following outcomes: (1) atherosclerotic vascular event (myocardial infarction, coronary death, unstable angina, ischemic heart failure, stroke, transient ischemic attack, arterial revascularization, or other peripheral arterial disease event), (2) nonatherosclerotic vascular event (other cardiac death, nonischemic heart failure, arrhythmia, or valvular heart disease event), and (3) any (atherosclerotic or nonatherosclerotic) vascular event (further details available in Item S1, provided as online supplementary material). The current analyses also include analyses of new diagnoses of cancer (excluding nonfatal nonmelanoma skin cancers) and cause-specific mortality. Among patients not on dialysis therapy at baseline, the main kidney disease outcomes included progression to ESRD (ie, transplantation or initiation of maintenance dialysis therapy) and the 2 composite outcomes of (1) ESRD or death and (2) ESRD or doubling in creatinine level. In addition, local hospital creatinine measurements allowed for the estimation of each individual’s annual rate of change in estimated GFR (eGFR) over time (as described previously).^16^

### Statistical Analysis

The etiologic relevance of baseline smoking status (current or former as compared to never smoker status) to particular diseases was estimated using Cox proportional hazards regression, with the proportional hazard assumption tested through examination of the time dependency of the Schoenfeld partial residuals. (For all outcomes, there was no good evidence that average relative risks [RRs] varied with increasing follow-up.) Analyses were adjusted for age, sex, ethnicity (white, black, Asian, and other), country, highest educational achievement (university, secondary school, vocational qualification, primary school or no formal education, and not specified), self-reported vascular disease (history of coronary heart, cerebrovascular, or peripheral arterial disease), and self-reported diabetes. Such adjustments were predicated on specific assumptions about the causal relationships between these variables, smoking status, and outcome (Fig S1). (There were no missing data for smoking status.) Anthropometric and biochemical measurements (including eGFR and urine albumin-creatinine ratio [UACR]) were not included in regression models because it was thought that they would be unlikely to modify a person’s smoking status (but could potentially be modified by it). In figures, the RR (approximated by the hazard ratio [HR] estimates from the Cox models) for each smoking group is accompanied by a group-specific confidence interval (CI) derived only from the variance of the log risk in that one category, hence associating each RR, including that for the reference group, with a group-specific CI that can be thought of as reflecting the amount of data only in that one category.^17^ Throughout the text, all quoted RRs are provided with the CI for the comparison with the specified reference group. Sensitivity analyses used Fine and Gray regression methods to take account of the potential for competing risks (ie, smoking causing death before the development of ESRD).^18^

## Results

### Characteristics of Study Population by Smoking Status

At baseline, 1,243 (13%) participants were current smokers, 3,272 (35%) were former smokers, and 4,755 (51%) were never smokers. Of current smokers, 475 (38%) reported smoking fewer than 10 cigarettes per day; 442 (36%), 10 to 19 cigarettes per day; and 326 (26%), 20 or more cigarettes per day. Median reported consumption was 10 cigarettes per day. Compared with never smokers, current smokers were younger and more likely to be male and white (Table 1). Proportions with prior vascular disease and in each education category in Table 1 are no longer adjusted for age, sex and ethnicity. Current smokers were also more likely than never smokers to have prior vascular disease, be a current drinker, and have lower educational achievement. Mean blood pressures (and use of antihypertensive treatments [Table S1]) were similar across the smoking groups, but mean body mass index was slightly lower in current compared with never smokers. Proportions of participants on dialysis therapy and with various underlying renal diagnoses were similar between the smoking groups (except that a higher proportion of participants with renovascular or hypertensive kidney disease were current smokers; Table S1). Although there were no significant differences in eGFRs, UACRs for those not on dialysis therapy were significantly higher among current smokers compared with former or never smokers (geometric mean UACRs of 234 ± 16 [estimated standard error], 172 ± 7, and 161 ± 6 mg/g, respectively; *P* < 0.001). Among 6,245 (67%) participants not on dialysis therapy at baseline, 88 (1%) had CKD stage 2; 2,155 (35%), stage 3; 2,565 (41%), stage 4; and 1,219 (20%), stage 5 at baseline (and 218 [3%] did not have a baseline creatinine value). Of 3,025 participants on dialysis therapy, 2,528 (84%) were receiving maintenance hemodialysis.

### Vascular Events

**Figure 1:**
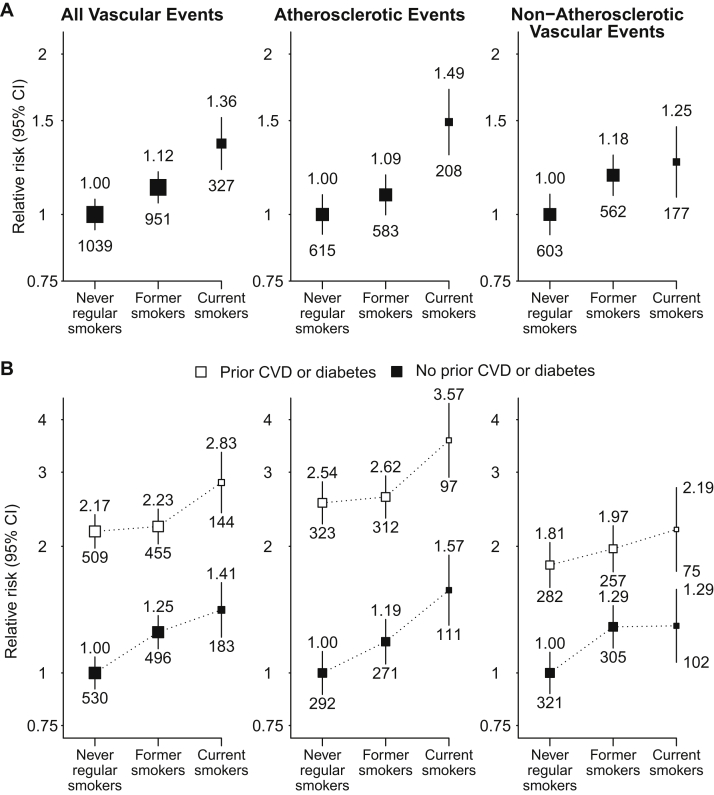
Relevance of baseline smoking status to vascular outcomes, (A) overall and (B) separately by history of cardiovascular disease (CVD) or diabetes. Relative risks are adjusted for age, sex, ethnicity, country, education, and for (A) only, prior disease (prior CVD and diabetes) and are quoted above the squares. Numbers of events in each group are quoted below the squares. Abbreviation: CI, confidence interval.

There were 138 (1.5%) and 204 (2.2%) participants who had less than 4 years of follow-up for mortality and morbidity outcomes, respectively. During a median of 4.9 years’ follow-up among survivors, 2,317 participants had at least 1 vascular event (annual rate, 66/1,000 patients per year), of whom 1,406 had an atherosclerotic event (39/1,000 per year) and 1,342 had a nonatherosclerotic event (36/1,000 per year). Compared with never smokers, vascular event risk was 12% (adjusted HR, 1.12; 95% CI, 1.02-1.24) higher among former smokers and 36% (adjusted HR, 1.36; 95% CI, 1.19-1.55) higher among current smokers, respectively, the latter reflecting significant increases in both atherosclerotic and nonatherosclerotic events (adjusted HRs of 1.49 [95% CI, 1.26-1.76] and 1.25 [95% CI, 1.05-1.50], respectively; Fig 1A). RRs associated with current smoking were similar among patients with and without a history of diabetes or vascular disease, so that the absolute excess risks attributed to smoking (overall, 25 [95% CI, 13-36] additional events per 1,000 per year) were about twice as large among those with such a history compared with those without (36 vs 18 additional events per 1,000 per year; Fig 1B; Table S2).

### Cancer

**Figure 2:**
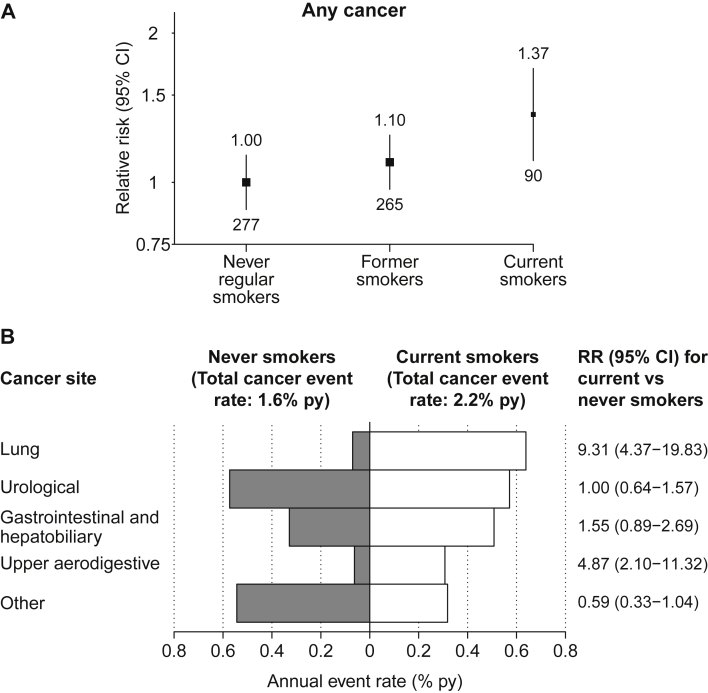
Relevance of baseline smoking status to (A) cancer incidence and (B) site-specific cancer. All relative risks (RRs) and annual event rates are adjusted for age, sex, ethnicity, country, education, and prior disease (prior cardiovascular and diabetes). In (A), RRs are quoted above the squares with numbers of events quoted below the squares. Abbreviations: CI, confidence interval; py, per year.

Overall, 632 participants (17/1,000 per year) developed a new cancer (fatal or nonfatal, excluding nonfatal nonmelanoma skin cancer) during follow-up. Compared with never smokers, former and current smokers had 10% (RR, 1.10; 95% CI, 0.92-1.31) and 37% (RR, 1.37; 95% CI, 1.07-1.76) increased risk for cancer, respectively (Fig 2A). Among current smokers, RRs were biggest for lung cancer at 9.31 (95% CI, 4.37-19.83) and upper aerodigestive tract cancers at 4.87 (95% CI, 2.10-11.32). Compared with never smokers, the absolute excess of cancer was 6 (95% CI, 1-11) additional cases per 1,000 per year (Fig 2B).

### Progression of CKD

**Figure 3:**
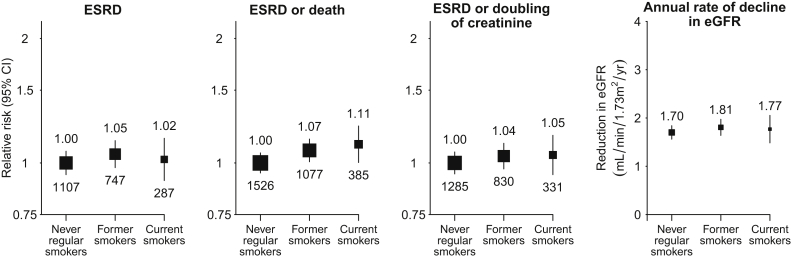
Relevance of baseline smoking status to renal progression among 6,245 patients not on dialysis therapy at randomization. Relative risks are adjusted for age, sex, ethnicity, country, education, and prior disease (prior cardiovascular and diabetes) and are quoted above the squares with the number of events quoted below the squares. Abbreviations: CI, confidence interval; eGFR, estimated glomerular filtration rate; ESRD, end-stage renal disease.

Of 6,245 participants who were not dialysis dependent at baseline, 2,141 developed ESRD during follow-up (100/1,000 per year). Compared with never smokers, former and current smokers had a similar rate of progression to ESRD (Fig 3). Sensitivity analyses using competing-risks methodology did not change any of the results materially (Fig S2). Similar patterns were observed for ESRD or death and for ESRD or doubling of serum creatinine level. Annual rates of change in kidney function prior to reaching ESRD among those with such assessments over a period of at least 1 year were also similar among current, former, and never smokers (Fig 3). Although there was an inverse association between baseline eGFR and rate of change in eGFR (ie, participants starting with higher eGFRs tended to progress more slowly), rates of change in eGFRs (and rates of ESRD) were similar in the 3 smoking categories at each starting eGFR (Figs S3 and S4). This was also true at different UACRs (Figs S3 and S4), different baseline levels of blood pressure or antihypertensive use (Fig S5), and for different causes of kidney disease (Fig S6). After adjustment for age, sex, ethnicity, and baseline UACR, there was no significant difference in UACRs taken in samples at 2.5 years (from participants who had not progressed to ESRD beforehand: geometric means of 158 [95% CI, 143-174] mg/g among never smokers and 166 [95% CI, 147-186] mg/g among current smokers).

### Mortality

**Figure 4:**
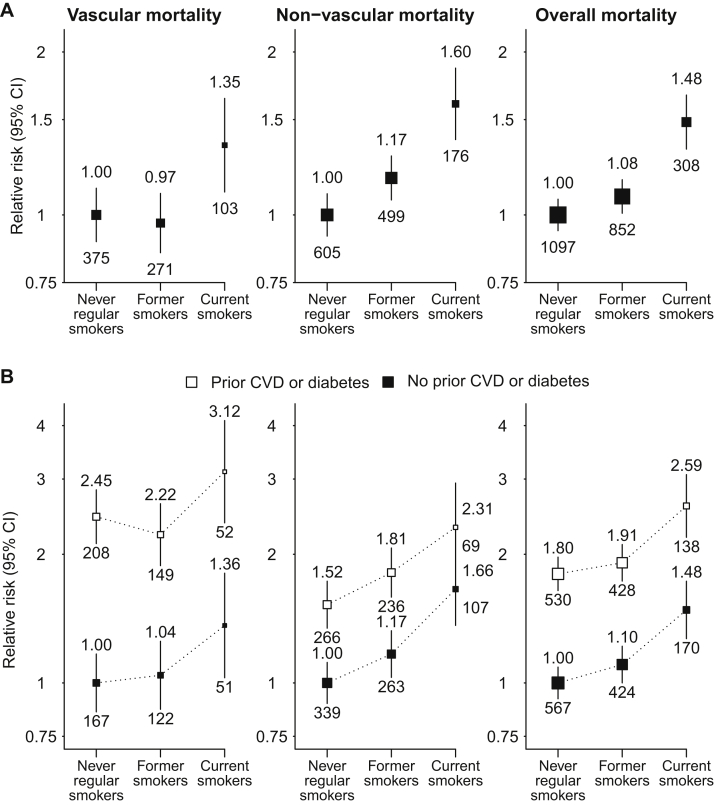
Relevance of baseline smoking status to cause-specific mortality, (A) overall and (B) separately by history of cardiovascular disease (CVD) or diabetes. Relative risks are adjusted for age, sex, ethnicity, country, education, and for (A) only, prior disease (prior CVD and diabetes) and are quoted above the squares. Numbers of events in each group are quoted below the squares. Abbreviation: CI, confidence interval.

A total of 2,257 participants died during follow-up, including 749 from a vascular cause (19/1,000 per year), 1,280 from a nonvascular cause (32/1,000 per year), and 228 (6/1,000 per year) from an unknown cause. Compared with never smokers, all-cause mortality rates were 8% (RR, 1.08; 95% CI, 0.98-1.19) higher among former smokers and 48% (RR, 1.48 [95% CI, 1.30-1.70]; absolute excess, 28 [95% CI, 17-38] per 1,000 per year) higher among current smokers. Among current smokers, significant increases were seen for both vascular (RR, 1.35 [95% CI, 1.07-1.69]; absolute excess, 7 [95% CI, 1-13] per 1,000 per year) and nonvascular (RR, 1.60 [95% CI, 1.34-1.91]; absolute excess, 19 [95% CI, 10-27] per 1,000 per year) causes of death (Fig 4). RRs associated with smoking were similar in participants with and without vascular disease or diabetes, but absolute excess risks were again about twice as high among participants with such prior disease. The increased risk for nonvascular mortality among current smokers chiefly reflected significant increases in deaths due to cancer (246 events; RR, 2.32 [95% CI, 1.58-3.40]; absolute excess, 7 [95% CI, 3-11] per 1,000 per year) and respiratory disease (224 events; RR, 2.25 [95% CI, 1.51-3.35]; absolute excess, 7 [95% CI, 2-11] per 1,000 per year).

### Sensitivity Analyses

RR estimates were not materially affected by additional adjustment for all baseline characteristics listed in Table 1 (rather than only the characteristics thought to be confounders: Fig S1), with the exception of ESRD, for which the additional adjustment for UACR resulted in an apparent protective association being observed between current smoking and ESRD risk (HR, 0.85; 95% CI, 0.74-0.98).

## Discussion

In this cohort of patients with CKD, smoking increased the risk for cardiovascular disease, cancer, and mortality, but was not associated with more rapid progression of kidney disease. These results are important because individuals with CKD are already at substantially increased risk for a wide variety of diseases, including cardiovascular disease,^8^ cancer,^7^ progression of kidney disease,^9^ and death.^8^ Although studies in the general population have already demonstrated that smoking increases the risks for many of these (in particular cardiovascular disease, cancer, and death^2^, ^3^, ^4^, ^5^), previous studies of people with CKD have been much more limited (eg, by their size and inadequate phenotyping of outcomes: Tables S3 and S4). By contrast, SHARP involves a large number of carefully adjudicated events and therefore provides a unique opportunity to directly assess the effects of smoking among patients with moderate to advanced CKD.

The current study has shown that smoking increases the risk for both atherosclerotic and nonatherosclerotic vascular disease (the latter being the predominant form of vascular disease in advanced CKD).^19^ In a previous study of 3,941 dialysis patients, smoking was associated with an increased risk for heart failure, but not with an increase in risk for atherosclerotic events (defined as ischemic heart or cerebrovascular diseases).^10^ However, unlike in SHARP, clinical outcomes were not adjudicated and the number of outcomes was small. In another study of 3,006 patients with CKD, smoking was associated with an increased risk for atherosclerotic vascular events, but the association with nonatherosclerotic outcomes was not reported separately.^13^ Among studies in the general population, 2 have reported associations between smoking and increased risk for atherosclerotic events among patients with mild to moderate CKD. In the first, among 1,249 patients with CKD (mean eGFR, 50 mL/min/1.73 m^2^), the RR for current versus never smoking was 1.82 (95% CI, 1.27-2.60).^20^ In the second study, among 807 patients with eGFRs < 60 mL/min/1.73 m^2^, the RR for current versus never smoking of a major coronary event (nonfatal myocardial infarction, coronary death, or revascularization) was 1.65 (95% CI, 1.01-2.67).^21^ The observed RR for current versus never smoking of any atherosclerotic vascular event in SHARP was similar to these estimates, at 1.49 (95% CI, 1.26-1.76), but somewhat less than that seen in general studies in which the risk for myocardial infarction is 2- to 5-fold higher among current than never smokers.^22^, ^23^ There are a number of possible explanations for this. First, in advanced CKD (especially among patients on dialysis therapy), the diagnosis of myocardial infarction is complicated by increased troponin concentrations and electrocardiographic changes in the absence of acute infarction, which may lead to underestimation of the magnitude of associations.^19^ Second, current smokers enrolled in SHARP had relatively low cigarette consumption (average, 10 cigarettes per day). Third, the average age of SHARP participants was 62 years and the RR associated with smoking decreases with age (for myocardial infarction, from ∼6 at age 30-39 years to 2.5 at age 60-69 years).^22^ Finally, smoking status was only assessed at baseline; the tendency for current smokers to quit during follow-up would be expected to have resulted in flattening of the observed risk relationships.^24^ These factors could also explain the somewhat weaker associations seen for mortality in SHARP when compared with those seen in the general population.

Patients with CKD are at increased risk for certain types of cancer,^7^, ^25^, ^26^ but to our knowledge, SHARP is the first study to investigate the effects of smoking on site-specific cancer among patients with CKD. As expected,^2^, ^5^ the cancers most strongly associated with smoking were lung and upper aerodigestive tract cancers, but the observed associations were still weaker than those seen in the general population.^5^

Our finding of an apparent lack of association between smoking and progression of kidney disease in a population with CKD is in contrast to previous studies of smoking among people with CKD (Table S4)^12^, ^13^ and is surprising in view of the evidence that smoking increases urinary albumin excretion^27^ and blood pressure^28^ and has adverse effects on intrarenal hemodynamics, particularly among patients with CKD.^29^ Although the distribution of particular causes of kidney disease in SHARP differed from those in previous studies, we found no evidence that cause of kidney disease significantly modified the relationship between smoking status and kidney disease progression (*P* = 0.2; Fig S6). The reason for the discrepancy between SHARP and other studies is therefore unclear, but is unlikely to be due to a lack of statistical power; SHARP included 2,000 ESRD events (more than all previous studies combined) and had >80% power [at 2p=0.05] to detect a RR of 1.10 for ESRD. One difference between our analyses and those of most previous studies is that we deliberately did not adjust for albuminuria because we thought it would be unlikely to influence a person’s decision to smoke (ie, it is unlikely to be a confounder). However, urinary albumin excretion may be modified by smoking,^27^, ^30^, ^31^ in which case adjustment for it could lead to the introduction of bias in the estimate of the association between smoking and kidney disease progression.^32^ Including albuminuria in our model creates what we believe to be an artificial association between smoking and reduced risk for ESRD, which is unlikely to reflect a true protective effect.

Our findings are of substantial public health importance because CKD and smoking are common in many populations. In the United States, for instance, 13% of the population is estimated to have CKD stages 1 to 4,^33^ and the proportion is similar in other countries.^34^, ^35^ Applying our RR estimates to national smoking prevalence estimates in populations with CKD,^10^, ^36^, ^37^ the proportion of all deaths attributable to smoking would be ∼6% and 7% in contemporary US and UK CKD populations, respectively, and ∼9% in people with CKD in China (where smoking is more common). Alternatively, the RR of 1.5 for all-cause mortality suggests that about one-third of all smokers with CKD would be killed by their habit. As in the general population, the key to avoiding most of this excess risk is likely to be cessation.^2^, ^4^, ^38^ In SHARP, the median age at CKD diagnosis was 52 years, so cessation at diagnosis would likely have avoided most of the smoking-attributable excess risk. Among smokers with CKD, quitting would be expected to reduce mortality and morbidity more than any currently available pharmacologic treatment.

Although large and prospective, our analyses have some limitations. First, we did not assess smoking status during follow-up and therefore were unable to adjust associations for the effects of changes in smoking status over time. As a consequence, we may have underestimated the strength of the association between continuing to smoke and adverse health outcomes.^39^ It also means that we were unable to assess directly the effects of smoking cessation, though the intermediate levels of risk observed for former smokers in our analyses are consistent with a strong beneficial effect of cessation existing that is similar in magnitude to that seen in other well-studied populations.^2^, ^4^, ^38^ Second, we did not collect detailed information about other aspects of smoking exposure (eg, age at starting, years since quitting, types of cigarette smoked, and inhalation behavior), somewhat limiting the range of analyses possible. Finally, as a special case of “collider bias,”^32^ it is possible that the deliberate selection into SHARP of patients with CKD might have created an inverse association between smoking status and other conditions (eg, cardiovascular disease) that share risk factors with CKD, potentially leading to a reduced association between smoking and those conditions.

In conclusion, smoking significantly increases the risks for vascular and nonvascular morbidity and for mortality in patients with CKD. For patients with CKD who smoke, the potential benefit from cessation would be substantial and certainly much larger than any drug treatment that is used for cardiovascular or nephroprotection. These benefits have not been properly appreciated by nephrologists and primary care physicians, and there is a need for smoking cessation programs to be established as pivotal components of the care of patients with CKD.

## Figures and Tables

**Table 1:** Baseline Demographics by Smoking Categories

	Current Smoker (n = 1,243)	Former Smoker (n = 3,272)	Never Smoker^a^ (n = 4,755)	*P*^b^
Age at randomization, y	57 ± 11	64 ± 11	61 ± 12	<0.001
Male sex	900 (72%)	2,445 (75%)	2,455 (52%)	<0.001
Ethnicity				<0.001
White	925 (74%)	2,769 (85%)	2,952 (62%)	
Black	58 (5%)	80 (2%)	126 (3%)	
Asian	198 (16%)	330 (10%)	1,558 (33%)	
Other	62 (5%)	93 (3%)	119 (3%)	
Education				<0.001
University	81 (7%)	366 (11%)	615 (13%)	
Secondary school	430 (35%)	1,017 (31%)	1,578 (33%)	
Vocational qualifications	299 (24%)	868 (27%)	960 (20%)	
Primary school or no formal education	246 (20%)	529 (16%)	950 (20%)	
Not specified	187 (15%)	492 (15%)	652 (14%)	
Prior vascular disease	208 (17%)	634 (19%)	551 (12%)	<0.001
Diabetes	227 (18%)	736 (22%)	1,131 (24%)	<0.001
Other baseline characteristics^c^				
Systolic BP, mm Hg	140 ± 22	139 ± 22	139 ± 22	0.2
Diastolic BP, mm Hg	80 ± 12	79 ± 13	79 ± 13	0.001
Body mass index, kg/m^2^	25.7 ± 5.4	27.5 ± 5.5	27.2 ± 5.5	<0.001
Current drinker	29%	30%	22%	<0.001
Kidney function				0.2
eGFR, mL/min/1.73 m^2^^d^	27.3 ± 12.9	26.6 ± 13.2	26.4 ± 13.1	
eGFR category^d^				
≥60 mL/min/1.73 m^2^	2%	1%	<0.5%	
≥30-<60 mL/min/1.73 m^2^	23%	25%	24%	
≥15-<30 mL/min/1.73 m^2^	29%	29%	28%	
<15 mL/min/1.73 m^2^	13%	14%	14%	
Receiving dialysis	33%	31%	33%	
UACR^c^^,^^d^				
Geometric mean, mg/g^e^	234 ± 16	172 ± 7	161 ± 6	<0.001
Median, mg/g	325 [84-1,074]	171 [37-654]	207 [43-789]	
UACR category^c^^,^^d^				
<30 mg/g	14%	19%	22%	
30-300 mg/g	36%	39%	38%	
>300 mg/g	50%	41%	41%	

*Note:* Unless otherwise indicated, values for categorical variables are given as number (percentage); values for continuous variables, as arithmetic mean ± standard deviation or median [interquartile range].

Abbreviations: BP, blood pressure; eGFR, estimated glomerular filtration rate; UACR, urinary albumin-creatinine ratio.

a Never regular smoker.

b *P* value for test of heterogeneity among the 3 smoking categories.

c Adjusted for age, sex, ethnicity, education, prior vascular disease, and prior diabetes.

d Among those not on dialysis therapy at randomization.

e Geometric mean ± approximate standard error.

## Supplementary Material

Supplementary Table S1 (PDF) Baseline characteristics and laboratory measures by smoking groups.

Supplementary Table S2 (PDF) Event rates among current and never smokers with and without prior diagnosis of CVD and/or diabetes.

Supplementary Table S3 (PDF) Summary of literature on relevance of smoking to cardiovascular morbidity and mortality.

Supplementary Table S4 (PDF) Summary of literature on relevance of smoking to development and progression of CKD.

Supplementary Figure S1 (PDF) Causal diagram of assumed associations between baseline smoking status, outcomes, and baseline characteristics.

Supplementary Figure S2 (PDF) Relevance of baseline smoking status to kidney disease progression in patients not receiving dialysis at randomization.

Supplementary Figure S3 (PDF) Relevance of baseline smoking status to annual eGFR decline, by baseline eGFR and UACR, in patients not receiving dialysis at randomization.

Supplementary Figure S4 (PDF) Relevance of smoking to ESRD by baseline eGFR and UACR.

Supplementary Figure S5 (PDF) Relevance of smoking to ESRD by baseline antihypertensive use and systolic blood pressure.

Supplementary Figure S6 (PDF) Relevance of baseline smoking status to ESRD by cause of kidney disease.

Supplementary Item S1 (PDF) Definitions of atherosclerotic and nonatherosclerotic vascular events.
