# Supplementary material for: Smoking and Adverse Outcomes in Patients With CKD: The Study of Heart and Renal Protection (SHARP)
Source: Am J Kidney Dis. 2016 Sep;68(3):371–80. doi: 10.1053/j.ajkd.2016.02.052 (PMC4996629; doi:10.1053/j.ajkd.2016.02.052)
Supplement: Supplementary Table S1 (PDF) — Baseline characteristics and laboratory measures by smoking groups. [file mmc1.pdf]

Table S1: Baseline characteristics and laboratory measures by smoking groups

	Smoking category			
	**Current smoker (n=1243)**	**Former smoker (n=3272)**	**Never regular smoker (n=4755)**	**P-value‡**
Total cholesterol (mg/dL)	186 (45)	189 (46)	190 (46)	0.1
LDL cholesterol (mg/dL)	105 (34)	107 (34)	108 (34)	0.004
HDL cholesterol (mg/dL)	44 (13)	43 (13)	43 (13)	0.02
Triglycerides (mg/dL)	207 (154)	212 (156)	201 (156)	0.02
Phosphate (mg/dL)	4.57 (1.48)	4.40 (1.52)	4.36 (1.46)	<0.001
Hemoglobin (g/dL)	12.33 (1.68)	12.14 (1.72)	12.16 (1.65)	0.003
Co-medication				
Antiplatelet therapy	26%	23%	22%	0.008
Oral anticoagulant therapy	2%	4%	3%	0.03
ACE inhibitor or ARB	54%	54%	55%	0.6
Beta blocker	39%	38%	38%	0.8
Calcium channel blocker	42%	42%	41%	0.7
Diuretic	42%	43%	40%	0.06
Erythropoiesis stimulating agent	26%	28%	27%	0.3
Sevelamer	9%	8%	8%	0.3
Renal diagnosis†				0.005
Glomerulonephritis	14%	18%	18%	
Diabetic nephropathy	16%	16%	13%	
Hypertensive/renovascular	20%	15%	17%	
Cystic kidney disease	10%	11%	12%	
Pyelonephritis	7%	7%	7%	
Other known cause	22%	20%	19%	
Unknown cause	12%	14%	13%	
Randomized to simvastatin plus ezetimibe	50%	50%	50%	0.9

Note: Mean (SD) or % shown, adjusted for age, sex, ethnicity, education, prior vascular disease and prior diabetes. Conversion factors for units: cholesterol in mg/dL to mmol/L, x0.02586; triglycerides in mg/dL to mmol/L, x0.01129; phosphate in mg/dL to mmol/L, x0.3229. †Among those not on dialysis at randomization. ‡P value for test of heterogeneity between the three smoking categories.
