# Supplementary material for: Smoking and Adverse Outcomes in Patients With CKD: The Study of Heart and Renal Protection (SHARP)
Source: Am J Kidney Dis. 2016 Sep;68(3):371–80. doi: 10.1053/j.ajkd.2016.02.052 (PMC4996629; doi:10.1053/j.ajkd.2016.02.052)
Supplement: Supplementary Table S2 (PDF) — Event rates among current and never smokers with and without prior diagnosis of CVD and/or diabetes. [file mmc2.pdf]

Table S2: Estimated event rates (per 1000 per year) among current and never smokers with and without a prior diagnosis of CVD and/or diabetes.

	Overall			Prior history of CVD/diabetes			No prior history of CVD/diabetes		
	Rate (SE) among current smokers*	Rate (SE) among never smokers*	Absolute excess risk† (95% CI)	Rate (SE) among current smokers*	Rate (SE) among never smokers*	Absolute excess risk† (95% CI)	Rate (SE) among current smokers*	Rate (SE) among never smokers*	Absolute excess risk† (95% CI)
Vascular events	93 (5)	68 (2)	25 (13 to 36)	134 (8)	98 (4)	36 (19 to 52)	67 (4)	49 (2)	18 (10 to 26)
Cancer incidence	22 (2)	16 (1)	6 (1 to 11)	24 (3)	17 (1)	7 (1 to 12)	21 (2)	15 (1)	6 (1 to 11)
All-cause mortality	86 (5)	59 (2)	28 (17 to 38)	116 (7)	80 (3)	36 (22 to 50)	66 (4)	46 (2)	21 (12 to 29)
Vascular mortality	28 (3)	21 (1)	7 (1 to 13)	42 (4)	32 (2)	10 (1 to 19)	18 (2)	14 (1)	4 (0 to 8)
Non-vascular mortality	50 (4)	32 (1)	19 (10 to 27)	63 (5)	40 (2)	23 (13 to 34)	42 (3)	27 (1)	15 (9 to 22)
Cancer death	12 (2)	5 (1)	7 (3 to 11)	15 (3)	6 (1)	9 (3 to 14)	10 (2)	4 (1)	6 (2 to 9)
Respiratory death	12 (2)	5 (1)	7 (2 to 11)	16 (3)	7 (1)	9 (3 to 15)	9 (2)	4 (1)	5 (2 to 8)

Note: *Poisson regression was used to model the common linear dependence of the log annual event rate on baseline age, sex, ethnicity, country, education, prior vascular disease, prior diabetes and smoking status (current, former, never). Mean (SE) event rates (per 1000 per year) were then estimated by applying the regression coefficients back to the participant characteristics, but forcing the smoking status to be either current or never smokers, as appropriate (estimates by prior disease were constructed similarly).

†Absolute excess risks were calculated by subtracting estimated rates among never regular smokers from estimated rates among current smokers.
