# Supplementary material for: Smoking and Adverse Outcomes in Patients With CKD: The Study of Heart and Renal Protection (SHARP)
Source: Am J Kidney Dis. 2016 Sep;68(3):371–80. doi: 10.1053/j.ajkd.2016.02.052 (PMC4996629; doi:10.1053/j.ajkd.2016.02.052)
Supplement: Supplementary Table S3 (PDF) — Summary of literature on relevance of smoking to cardiovascular morbidity and mortality. [file mmc3.pdf]

Table S3: Summary of literature on relevance of smoking to cardiovascular morbidity and mortality

Author	Year	Design	Population	Mean eGFR	Reported consumption among current smokers	Outcome	N	Events	Average length of follow-up	Results (current vs not current smokers unless stated)	Additional covariates included in model
Grams[1]	2012	Prospective	CKD (39.7 % female)	39	Not stated	Pre-ESRD mortality (from any cause)	1722	243	Median 17.6 yrs	HR 1.5 (1.01-2.23)	Age, sex, ethnicity, SBP, cause of kidney disease, diabetes, presence of CVD, log 24-hour proteinuria, and eGFR.
Ricardo[2]	2015	Prospective	CKD (48% female)	43	Not stated	Atherosclerotic vascular events; all-cause mortality	3006	355; 437	Median 4 years	HR 1.82 (1.33-2.5); 2.22 (1.67-2.94)	Age, sex, ethnicity, education, diabetes, dyslipidemia, hypertension, any CVD, ACEi/ARB, eGFR and log 24-hour urine protein excretion.
Muntner[3]	2005	Prospective	CKD (67% female)		Not collected	CHD	807	108	Average 9 years	HR 1.65 (1.01-2.67)	Age, sex, ethnicity
Shlipak[4]	2005	Prospective	CKD (53% female)	50	Not reported	CV death	1249	342	Average 8.6 years	HR 1.82 (1.27-2.60)	Age, sex, ethnicity, education, diuretic use, prevalent CV disease
Foley[5]	2003	Prospective	Dialysis (47.2% female)	N/A	Not reported	Ischaemic heart disease; Congestive heart failure; cerebrovascular disease; peripheral vascular disease; all-cause mortality	3941	232; 297; 64; 383; 1179	Average 2.2 years	Not associated [HR not given]; HR 1.59 (1.16-2.17); Not associated [HR not given]; HR 1.68 (1.27-2.22); HR 1.37 (1.15-1.64)	Age, sex, ethnicity, primary renal disorder, prior CV disease, diabetic status, dialysis modality
Fishbane[6]	1996	Prospective	Dialysis (41% female)	N/A	Not reported	All-cause and CV mortality	132	28; 16	1 year	Smoking history present vs absent: HR 0.9 (0.5-2.1); HR 1.2 (0.4-2.2)	Age, sex, ethnicity, diabetes, hypertension, cholesterol, kt/V, serum albumin, prior vascular disease, AAI

Abbreviations:

2xCr = doubling of creatinine; 50%↓eGFR = halving of eGFR; AAI = ankle-arm blood pressure index; ACEi = angiotensin coverting enzyme inhibitor; ADPKD = autosomal dominant polycystic kidney disease; ARB = angiotensin receptor blocker; BMI = body-mass index; CCB = calcium channel blocker; CHD = coronary heart disease; CKD = chronic kidney disease; CVD = cardiovascular disease; DBP = diastolic blood pressure; DN = diabetic nephropathy; ESRD = end-stage renal disease; (e)GFR = (estimated) glomerular filtration rate; HR = hazard ratio; HDL-C = high density lipoprotein cholesterol; IgA = IgA nephropathy; IGT = impaired glucose tolerance; NSAID = non-steroidal anti-inflammatory drug; OR = odds ratio; PCR = protein creatinine ratio; SBP = systolic blood pressure

Works Cited:

1. Grams ME, Coresh J, Segev DL, Kucirka LM, Tighiouart H, Sarnak MJ. Vascular disease, ESRD, and death: interpreting competing risk analyses. *Clin J Am Soc Nephrol* 2012; **7** (10): 1606-1614
2. Ricardo AC, Anderson CA, Yang W, et al. Healthy lifestyle and risk of kidney disease progression, atherosclerotic events, and death in CKD: findings from the Chronic Renal Insufficiency Cohort (CRIC) Study. *Am J Kid Dis* 2015; **65** (3): 412-424
3. Muntner P, He J, Astor BC, Folsom AR, Coresh J. Traditional and nontraditional risk factors predict coronary heart disease in chronic kidney disease: results from the atherosclerosis risk in communities study. *J Am Soc Nephrol* 2005; **16** (2): 529-538
4. Shlipak MG, Fried LF, Cushman M, et al. Cardiovascular mortality risk in chronic kidney disease: comparison of traditional and novel risk factors. *J Am Med Assoc* 2005; **293** (14): 1737-1745
5. Foley RN, Herzog CA, Collins AJ. Smoking and cardiovascular outcomes in dialysis patients: The United States Renal Data System Wave 2 Study. *Kidney Int.* 2003; **63** (4): 1462-1467
6. Fishbane S, Youn S, Flaster E, Adam G, Maesaka JK. Ankle-arm blood pressure index as a predictor of mortality in hemodialysis patients. *Am J Kid Dis* 1996; **27** (5): 668-672
