# Supplementary material for: Smoking and Adverse Outcomes in Patients With CKD: The Study of Heart and Renal Protection (SHARP)
Source: Am J Kidney Dis. 2016 Sep;68(3):371–80. doi: 10.1053/j.ajkd.2016.02.052 (PMC4996629; doi:10.1053/j.ajkd.2016.02.052)
Supplement: Supplementary Table S4 (PDF) — Summary of literature on relevance of smoking to development and progression of CKD. [file mmc4.pdf]

Table S4: Summary of literature on relevance of smoking to development and progression of CKD

Author	Year	Design	Population	Mean eGFR	Reported consumption among current smokers	Outcome	N	Events	Average length of follow-up	Results (current vs not current smokers unless stated)	Additional covariates included in model
Yoshida[1]	2008	Prospective	CKD		Not stated	Slope of 1/Cr	2012	-		-2.06 ml/min; p=0.014	Age, sex, high BMI, metabolic syndrome, proteinuria, hypertension, high triglyceride, low HDL-C, high fasting plasma glucose, ACEi/ARB therapy, CCB therapy
Grams[2]	2012	Prospective	CKD	39	Not stated	ESRD	1722	1099	Median 17.6 yrs	HR 1.3 (1.09-1.55)	Age, sex, ethnicity, SBP, cause of kidney disease, diabetes, presence of CVD, log 24-hour proteinuria, and eGFR.
Tanaka[3]	2013	Prospective	CKD	55	Not stated	ESRD/2xCr	449	46	Median 3.3 years	HR 1.25 (0.41- 3.84)	Age, sex, diabetes, hypertension, dylipidemia, eGFR, proteinuria, serum albumin, skin autofluorescence
Ricardo[4]	2015	Prospective	CKD	43	Not stated	ESRD or 50%↓eGFR	3006	726	Median 4 years	HR 1.47 (1.19-1.82)	Age, sex, ethnicity, education, diabetes, dyslipidemia, hypertension, any CVD, ACEi/ARB, eGFR and log 24-hour urine protein excretion.
Haroun[5]	2003	Prospective	General		Not stated	CKD	23,534	143	18 years	HR 2.6 (1.8–3.7)	Age, sex, treated diabetes
Shankar[6]	2006	Prospective	General	n/a	Not stated	CKD	3392	114	5 years	HR: 1.93 (1.15-3.25) [current vs never smokers]	Age, sex, education, body mass index, current NSAID use, hypertension status, diabetes status, history of CVD, and heavy drinking.
Ishani[7]	2006	Prospective	General	n/a	Not stated	ESRD	12,866	213	25 years	HR 1.84 (1.35-2.51)	Age, black ethnicity, BMI, family history of diabetes, triglycerides, HDL-C, LDL-C, uric acid, fasting glucose, SBP, eGFR, haematocrit and urine proteinuria

**Author**	**Year**	**Design**	**Population**	**Mean eGFR**	**Reported consumption among current smokers**	**Outcome**	**N**	**Events**	**Average length of follow-up**	**Results (current vs not current smokers unless stated)**	**Additional covariates included in model**
Yamagata[8]	2007	Prospective	General	80	Not stated	CKD3-5	123,764	19,411	10 years	HR 1.13 (1.05-1.22) in men;<br><br>1.16 (1.06-1.26) in women	Age, proteinuria, hematuria, concomitant proteinuria and hematuria, IGT, diabetes, hypertension, hypercholesterolemia, low HDL-C, hypertriglyceridemia, obesity,<br><br>alcohol consumption
Yacoub[9]	2010	Case-control	General	n/a	Not stated	CKD	569	198		OR 1.63 (1.08-2.45) [current vs never smokers]	Age and sex
Hallan[10]	2011	Prospective	General	97	Average 14.5 pack years	CKD5	65,589	124	Median 10.3 yrs	HR 4.01 (1.43-11.25) in <70;<br><br>1.09 (0.51-2.33) in ≥70	Age, sex, high education, physical inactivity, diabetes, prevalent CVD, antihypertensive use, SBP, waist circumference, total/HDL-C, eGFR, ACR
Lipworth[11]	2012	Prospective	General	n/a	Not stated	ESRD	79,943	662	4 yrs	HR 1.2 (1.02-1.4)	Age, sex, recruitment source, education, annual household income, history of diabetes,<br><br>hypertension,<br><br>stroke, high cholesterol, and MI/CABG.
Carter[12]	2015	Prospective	General		Cigarettes smoked per day:<br><br>Men 22.6% <10; 39.8% ≥ 20<br><br>Women 40.7% <10; 20.9% ≥20	Renal death	954,029	1072	10 years	RR 2.1 (1.6-2.6) in men;<br><br>1.9 (1.5-2.5) in women	Age, race, educational<br><br>level, current alcohol use, and cohort.
Paterson[13]	2005	Prospective	ADPKD	83	Average 18 pack years	Creatinine clearance	406	-		ns	Univariate analyses
Ozkok[14]	2013	Prospective	ADPKD	60	Average 4.2 pack years	>1 ml/min/yr ↓GFR	171	-	8 years	HR 0.78 (0.28-2.16)	Age, gender, baseline serum creatinine, presence of hypertension, abdominal wall hernia, hepatic cyst, familial history of ADPKD, macroscopic hematuria, 24-h proteinuria, urinary stone, palpable kidneys in physical examination and use of ACEIs and/or ARBs

Author	Year	Design	Population	Mean eGFR	Reported consumption among current smokers	Outcome	N	Events	Average length of follow-up	Results (current vs not current smokers unless stated)	Additional covariates included in model
Orth[15]	1998	Case-control	ADPKD/ IgA	n/a		ESRD	204	102		OR for >5 pack years 4.5 (1.9-10.9) in men; 1.0 (0.3-3.4) in women	None
Sawicki[16]	1994	Prospective	DN	n/a	Average 18 pack years	1.36 ml/min ↓GFR	93	25	1 year	OR 2.74 (1.57-4.81) per 10 pack years	Age, sex, diabetes duration, HbA1c, BMI, daily protein intake, 24-h urinary sodium excretion, SBP, DBP
Chuahirun[17]	2002	Prospective	DN	98	Not stated	Slope of eGFR	33	-	5 years	-0.59 to -0.09 ml/min (p=0.009)	Age, gender, ethnicity, initial mean BP, initial PCR
Phisitkul[18]	2008	Prospective	DN	95		Slope of 1/Cr	91	-		Not stated. p=0.041	Age, sex, ethnicity, diabetes, SBP, urine albumin, HbA1c, LDL-C, HDL-C, eGFR

Abbreviations:

2xCr = doubling of creatinine; 50%↓eGFR = halving of eGFR; ACEi = angiotensin coverting enzyme inhibitor; ADPKD = autosomal dominant polycystic kidney disease; ARB = angiotensin receptor blocker; BMI = body-mass index; CCB = calcium channel blocker; CKD = chronic kidney disease; CVD = cardiovascular disease; DBP = diastolic blood pressure; DN = diabetic nephropathy; ESRD = end-stage renal disease; (e)GFR = (estimated) glomerular filtration rate; HR = hazard ratio; HDL-C = high density lipoprotein cholesterol; IgA = IgA nephropathy; IGT = impaired glucose tolerance; NSAID = non-steroidal anti-inflammatory drug; OR = odds ratio; PCR = protein creatinine ratio; SBP = systolic blood pressure

Works Cited:

1. Yoshida T, Takei T, Shirota S, et al. Risk factors for progression in patients with early-stage chronic kidney disease in the Japanese population. *Int Med* 2008; **47** (21): 1859-1864

2. Grams ME, Coresh J, Segev DL, Kucirka LM, Tighiouart H, Sarnak MJ. Vascular disease, ESRD, and death: interpreting competing risk analyses. *Clin J Am Soc Nephrol* 2012; **7** (10): 1606-1614

3. Tanaka K, Nakayama M, Kanno M, et al. Skin autofluorescence is associated with the progression of chronic kidney disease: a prospective observational study. *PLoS One* 2013; **8** (12): e83799

4. Ricardo AC, Anderson CA, Yang W, et al. Healthy lifestyle and risk of kidney disease progression, atherosclerotic events, and death in CKD: findings from the Chronic Renal Insufficiency Cohort (CRIC) Study. *Am J Kid Dis* 2015; **65** (3): 412-424

5. Haroun MK, Jaar BG, Hoffman SC, Comstock GW, Klag MJ, Coresh J. Risk factors for chronic kidney disease: a prospective study of 23,534 men and women in Washington County, Maryland. *J Am Soc Nephrol* 2003; **14** (11): 2934-2941

6. Shankar A, Klein R, Klein BE. The association among smoking, heavy drinking, and chronic kidney disease. *Am J Epidemiol* 2006; **164** (3): 263-271

7. Ishani A, Grandits GA, Grimm RH, et al. Association of single measurements of dipstick proteinuria, estimated glomerular filtration rate, and hematocrit with 25-year incidence of end-stage renal disease in the multiple risk factor intervention trial. *J Am Soc Nephrol* 2006; **17** (5): 1444-52

8. Yamagata K, Ishida K, Sairenchi T, et al. Risk factors for chronic kidney disease in a community-based population: a 10-year follow-up study. *Kidney Int.* 2007; **71** (2): 159-66

9. Yacoub R, Habib H, Lahdo A, et al. Association between smoking and chronic kidney disease: a case control study. *BMC Public Health.* 2010; **10**: 731

10. Hallan SI, Orth SR. Smoking is a risk factor in the progression to kidney failure. *Kidney Int.* 2011; **80** (5): 516-23

11. Lipworth L, Mumma MT, Cavanaugh KL, et al. Incidence and predictors of end stage renal disease among low-income blacks and whites. *PLoS One* 2012; **7** (10): e48407

12. Carter CE, Katz R, Kramer H, et al. Influence of urine creatinine concentrations on the relation of albumin-creatinine ratio with cardiovascular disease events: the Multi-Ethnic Study of Atherosclerosis (MESA). *Am J Kid Dis* 2013; **62** (4): 722-9

13. Paterson AD, Magistroni R, He N, et al. Progressive loss of renal function is an age-dependent heritable trait in type 1 autosomal dominant polycystic kidney disease. *J Am Soc Nephrol* 2005; **16** (3): 755-62

14. Ozkok A, Akpinar TS, Tufan F, et al. Clinical characteristics and predictors of progression of chronic kidney disease in autosomal dominant polycystic kidney disease: a single center experience. *Clin Exp Nephrol* 2013; **17** (3): 345-51
15. Orth SR, Stockmann A, Conradt C, et al. Smoking as a risk factor for end-stage renal failure in men with primary renal disease. *Kidney Int.* 1998; **54** (3): 926-31
16. Sawicki PT, Didjurgeit U, Muhlhauser I, Bender R, Heinemann L, Berger M. Smoking is associated with progression of diabetic nephropathy. *Diabetes Care* 1994;**17** (2): 126-31
17. Chuahirun T, Wesson DE. Cigarette smoking predicts faster progression of type 2 established diabetic nephropathy despite ACE inhibition. *Am J Kid Dis* 2002; **39** (2): 376-82
18. Phisitkul K, Hegazy K, Chuahirun T, et al. Continued smoking exacerbates but cessation ameliorates progression of early type 2 diabetic nephropathy. *Am J Med Sci* 2008; **335** (4): 284-91
