# Supplementary material for: Smoking and Adverse Outcomes in Patients With CKD: The Study of Heart and Renal Protection (SHARP)
Source: Am J Kidney Dis. 2016 Sep;68(3):371–80. doi: 10.1053/j.ajkd.2016.02.052 (PMC4996629; doi:10.1053/j.ajkd.2016.02.052)
Supplement: Supplementary Figure S1 (PDF) — Causal diagram of assumed associations between baseline smoking status, outcomes, and baseline characteristics. [file mmc5.pdf]

Figure S1: Causal diagram showing assumed associations between baseline smoking status, outcomes and baseline characteristics

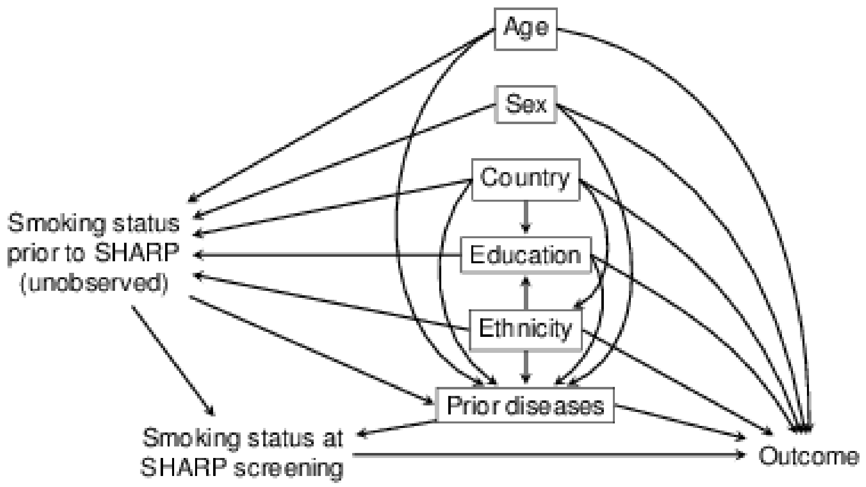

A participant's smoking status prior to SHARP is also assumed to be prior to any documented vascular disease and could therefore affect the risk of developing such disease. As prior vascular disease at the time of entry into SHARP would have been diagnosed prior to screening, then a participant's smoking status at screening could not influence the risk of prior disease but might have been modified by it (i.e. the participant quits smoking as a result of being diagnosed with vascular disease). Analyses were adjusted for the confounders enclosed by boxes in the causal diagram.
