# Supplementary material for: Smoking and Adverse Outcomes in Patients With CKD: The Study of Heart and Renal Protection (SHARP)
Source: Am J Kidney Dis. 2016 Sep;68(3):371–80. doi: 10.1053/j.ajkd.2016.02.052 (PMC4996629; doi:10.1053/j.ajkd.2016.02.052)
Supplement: Supplementary Figure S2 (PDF) — Relevance of baseline smoking status to kidney disease progression in patients not receiving dialysis at randomization. [file mmc6.pdf]

Figure S2: Relevance of baseline smoking status to renal progression among 6245 patients not on dialysis at randomization using Fine and Gray regression

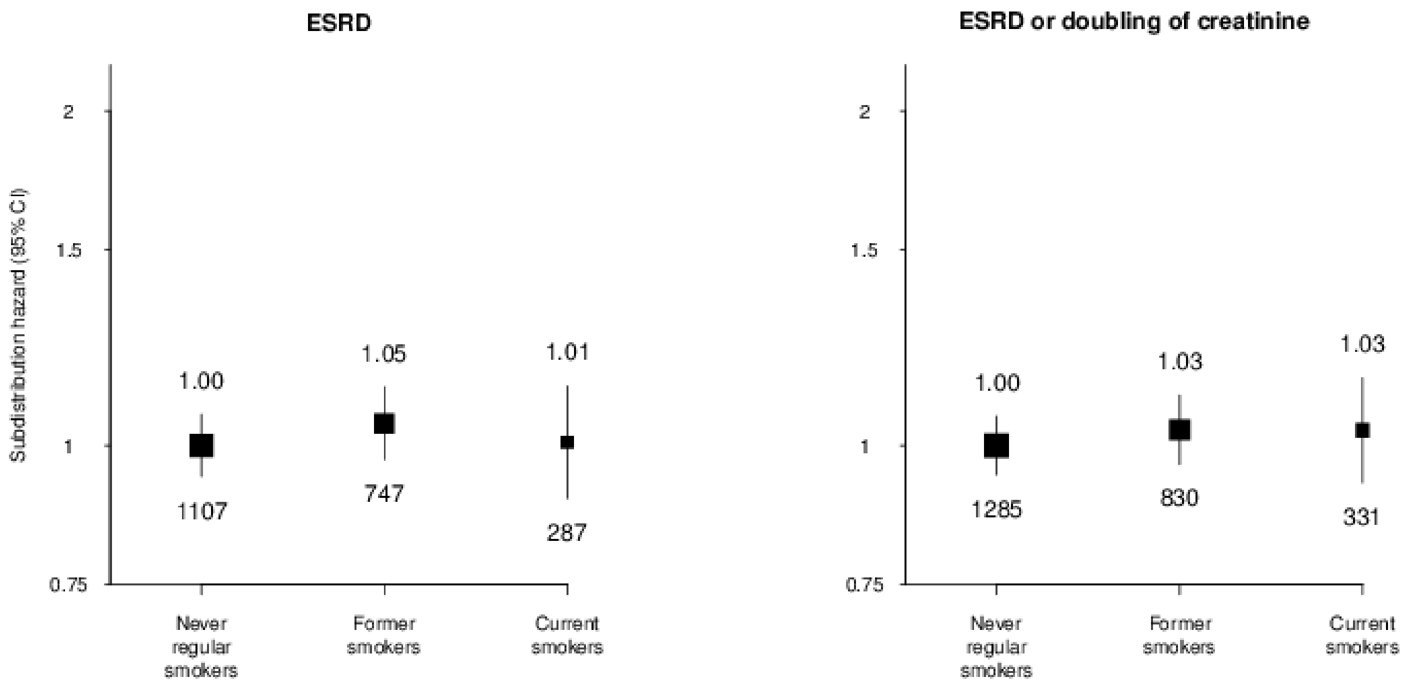

Subdivision hazards adjusted for age, sex, ethnicity, country, education, and prior disease. Fine and Gray regression was used to take account of the competing risk of death before ESRD. The estimated subdistribution hazards here reflect the actual rate at which current smokers would be expected to present with ESRD, whereas the Cox estimates previously presented (in Figure 3 of the article) reflect the rates in the hypothetical absence of deaths before ESRD.
