# Supplementary material for: Smoking and Adverse Outcomes in Patients With CKD: The Study of Heart and Renal Protection (SHARP)
Source: Am J Kidney Dis. 2016 Sep;68(3):371–80. doi: 10.1053/j.ajkd.2016.02.052 (PMC4996629; doi:10.1053/j.ajkd.2016.02.052)
Supplement: Supplementary Figure S3 (PDF) — Relevance of baseline smoking status to annual eGFR decline, by baseline eGFR and UACR, in patients not receiving dialysis at randomization. [file mmc7.pdf]

Figure S3: Relevance of baseline smoking status to annual rate of decline in eGFR, by baseline eGFR and baseline albumin:creatinine ratio, among 6245 patients not on dialysis at randomization

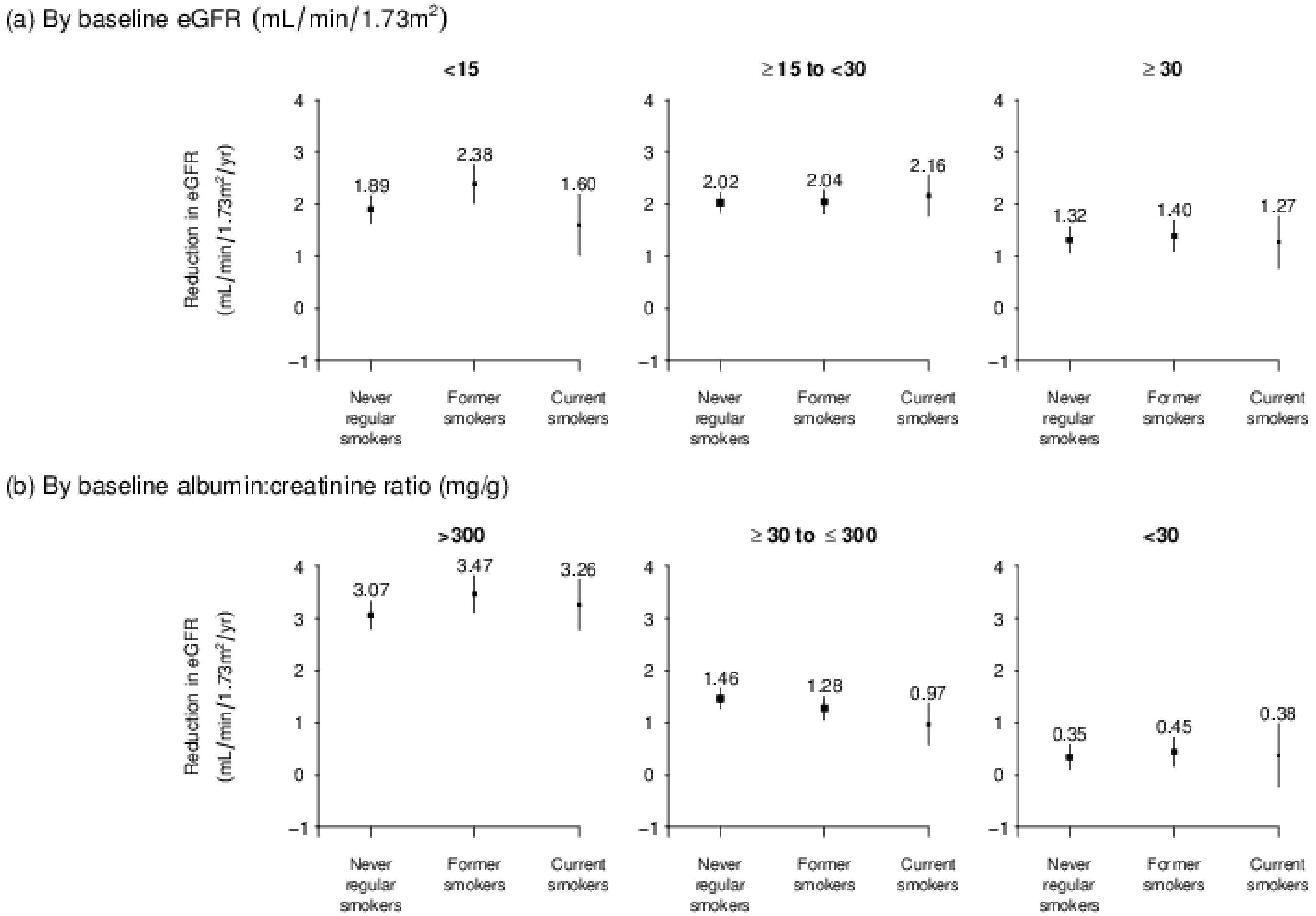

Rates adjusted for age, sex, ethnicity, country, education and prior disease (prior cardiovascular or diabetes).
