# Supplementary material for: Smoking and Adverse Outcomes in Patients With CKD: The Study of Heart and Renal Protection (SHARP)
Source: Am J Kidney Dis. 2016 Sep;68(3):371–80. doi: 10.1053/j.ajkd.2016.02.052 (PMC4996629; doi:10.1053/j.ajkd.2016.02.052)
Supplement: Supplementary Figure S4 (PDF) — Relevance of smoking to ESRD by baseline eGFR and UACR. [file mmc8.pdf]

Figure S4: Relevance of smoking to ESRD by (A) baseline eGFR and (B) albumin:creatinine ratio

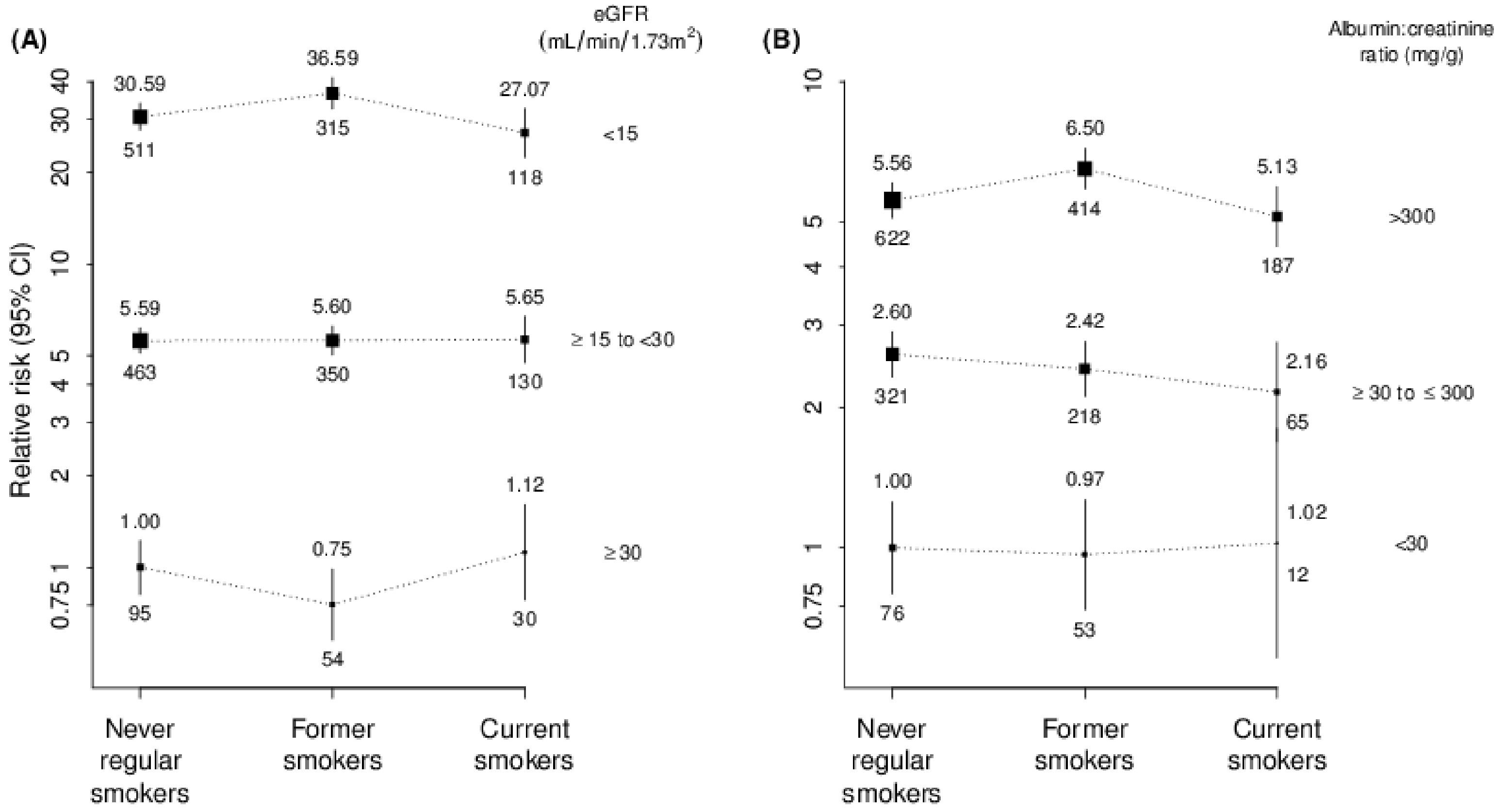

Relative risks adjusted for age, sex, ethnicity, country, education and prior disease (prior cardiovascular and diabetes). In panel (A), never regular smokers with eGFR greater than or equal to 30 used as reference category. In panel (B), never regular smokers with albumin:creatinine ratio <30 mg/g used as reference category.
