# Supplementary material for: Smoking and Adverse Outcomes in Patients With CKD: The Study of Heart and Renal Protection (SHARP)
Source: Am J Kidney Dis. 2016 Sep;68(3):371–80. doi: 10.1053/j.ajkd.2016.02.052 (PMC4996629; doi:10.1053/j.ajkd.2016.02.052)
Supplement: Supplementary Figure S5 (PDF) — Relevance of smoking to ESRD by baseline antihypertensive use and systolic blood pressure. [file mmc9.pdf]

Figure S5: Relevance of smoking to ESRD by (A) baseline antihypertensive use and (B) baseline systolic blood pressure

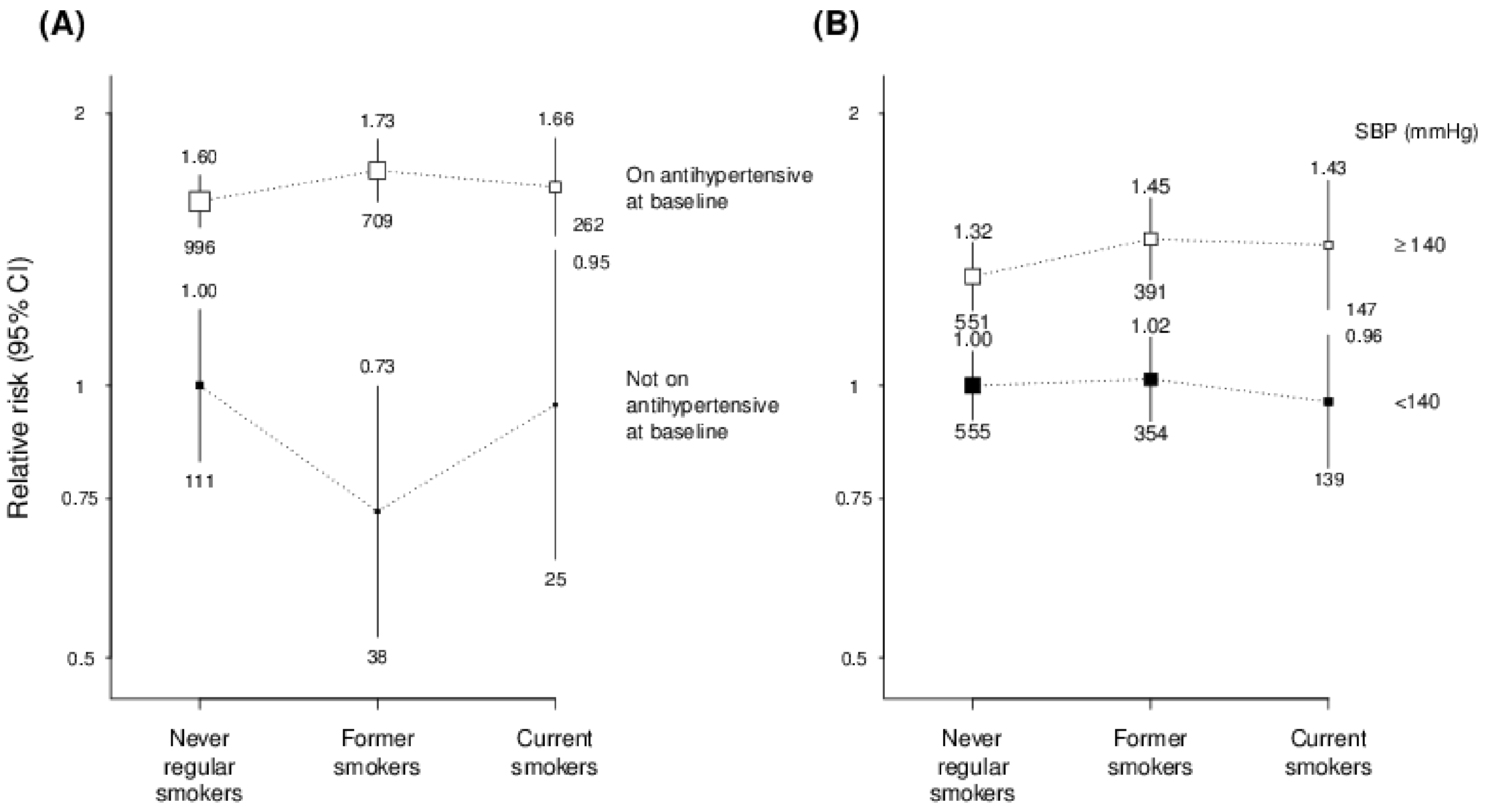

Relative risks adjusted for age, sex, ethnicity, country, education and prior disease (prior cardiovascular and diabetes). In panel (A), never regular smokers not on antihypertensive at baseline used as reference category. In panel (B), never regular smokers with systolic blood pressure <140 mmHg used as reference category
