# Supplementary material for: Smoking and Adverse Outcomes in Patients With CKD: The Study of Heart and Renal Protection (SHARP)
Source: Am J Kidney Dis. 2016 Sep;68(3):371–80. doi: 10.1053/j.ajkd.2016.02.052 (PMC4996629; doi:10.1053/j.ajkd.2016.02.052)
Supplement: Supplementary Figure S6 (PDF) — Relevance of baseline smoking status to ESRD by cause of kidney disease. [file mmc10.pdf]

Figure S6: Relevance of baseline smoking status to ESRD, by cause of kidney disease

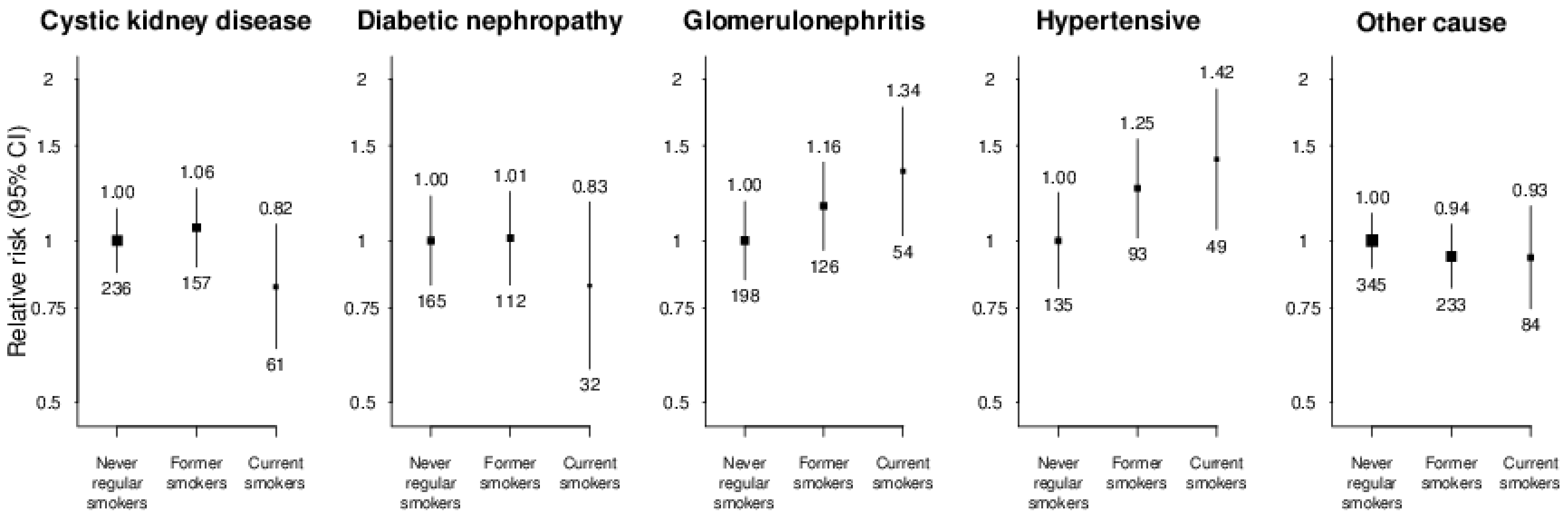

Relative risks adjusted for age, sex, ethnicity, country, education and prior disease. Test for interaction between smoking status and cause of kidney disease: χ^2_6=11.2; p=0.19.
