# Supplementary material for: Smoking and Adverse Outcomes in Patients With CKD: The Study of Heart and Renal Protection (SHARP)
Source: Am J Kidney Dis. 2016 Sep;68(3):371–80. doi: 10.1053/j.ajkd.2016.02.052 (PMC4996629; doi:10.1053/j.ajkd.2016.02.052)
Supplement: Supplementary Item S1 (PDF) — Definitions of atherosclerotic and nonatherosclerotic vascular events. [file mmc11.pdf]

Item S1: Definitions of atherosclerotic and non-atherosclerotic vascular events

Atherosclerotic vascular event

Major atherosclerotic event (MAE):

- Major coronary events: non-fatal myocardial infarction or coronary death
- Ischaemic stroke
- Arterial revascularization: coronary or non-coronary (excluding interventions on haemodialysis access)

Plus:

- Other atherosclerotic coronary events: unstable angina, heart failure related to ischaemic heart disease
- Other atherosclerotic cerebrovascular events: transient ischaemic attack, amaurosis fugax, retinal artery occlusion
- Other atherosclerotic peripheral arterial disease:
    - aortic aneurysm (including rupture and dissection)
    - limb ischaemia, limb artery embolism or thrombosis

Non-atherosclerotic vascular event

Non-atherosclerotic cardiac events:

- Non-coronary cardiac death
- Heart failure not related to ischaemic heart disease (hypertrophic cardiomyopathy, cor pulmonale)
- Arrhythmias: atrial fibrillation, ventricular tachycardia, other tachycardia, bradycardia/heart block, other arrhythmia, cardiac arrest
- Valvular heart disease (including valve repair or replacement)
- Pericardial disease: pericarditis, effusion

Other non-atherosclerotic events:

- Haemorrhagic stroke
- Subarachnoid haemorrhage
